# Readiness for interprofessional learning among undergraduate health discipline students: A multisite study

**DOI:** 10.4102/curationis.v49i1.2803

**Published:** 2026-03-26

**Authors:** Nestor Tomas, Lopes Markus

**Affiliations:** 1 Department of General Nursing Science, Faculty of Health Science and Veterinary Medicine, University of Namibia, Rundu, Namibia

**Keywords:** interprofessional learning, interprofessional education, patient care, readiness, students

## Abstract

**Background:**

The World Health Organization (WHO) recognises interprofessional learning as an effective component of transformative medical education. Health discipline students represent the core disciplines that form the primary collaborative healthcare team in clinical settings. However, the implementation of interprofessional learning within health disciplines in Namibia remains unassessed.

**Objectives:**

To assess and describe the readiness for interprofessional learning among undergraduate health discipline students at three campuses of the University of Namibia.

**Method:**

A quantitative descriptive online survey was conducted to recruit 236 health discipline students through purposive sampling. To allow enough time for data collection, data were collected between May and August 2023, using the validated readiness for Interprofessional Learning Scale.

**Results:**

The mean teamwork and collaboration were high at 4.22 ± 1.02, roles and responsibilities had a high score for interprofessional learning readiness at 1.93 ± 0.25, while the professional identity readiness domain was low at 1.40 ± 0.27. Professional identity showed a strong positive correlation with course of study (*rho* = 0.722; *p* = 0.010). Teamwork and collaboration (*R*^2^ = 0.838; *p* < 0.001) and roles and responsibilities (*R*^2^ = 0.208; *p* < 0.001) emerged as the most robust predictors of readiness, accounting for 20% and 83.8% of the variance in readiness.

**Conclusion:**

Using the framework for interprofessional education, the study assessed student readiness and determined that both teamwork and collaboration and roles and responsibilities were the major statistically significant predictors of interprofessional readiness.

**Contribution:**

This study identified predictors of interprofessional collaboration among health discipline students in Namibia.

## Introduction

The concept of interprofessional learning (IPL) is centred on improving teamwork among different health disciplines to ensure that patient care is of high quality and positive health outcomes are achieved (Reeves et al. 2010). The World Health Organization (WHO) defines IPL as the collaboration of two or more professions learning from one another, with the aim of promoting teamwork to enhance health outcomes and facilitate effective collaboration in future practice (Samarasekera et al. 2022; WHO 2020). Interprofessional learning readiness describes a student’s attitude and willingness to collaborate with peers from various health disciplines, forming a crucial foundation for effective interprofessional education, which, in turn, is essential for achieving successful, team-based patient care (Atwa et al. 2023). While IPL allows for the development of professional identities and a better understanding of other professions, as well as fostering a sense of belonging and teamwork (Filies & Frantz 2021), no research has been conducted on this topic among undergraduate health discipline students in Namibia.

### Background

Globally, numerous health issues require the involvement of a variety of healthcare and social workers, including generalists and specialists (Tong et al. 2020). The ageing population and increasing burden of chronic diseases have resulted in unique challenges for healthcare professionals, particularly in primary care (Khan, Addo & Findlay 2024; Sun & Li 2023). Literature reveals that healthcare has suffered fragmentation, leading to poor-quality care and higher health costs (Gatome-Munyua et al. 2025; Prior et al. 2023). Consequently, new care models have arisen, including those based on interprofessional collaboration, to improve healthcare efficiency and patient outcomes by reducing costs (Carron et al., 2021; Djaharuddin et al. 2025). Interprofessional collaborative practice has proven beneficial in delivering safe and high-quality patient care (Bouton et al. 2023). Additionally, it should be noted that IPL has the potential to enhance the clinical and medical knowledge and skills of upcoming health workers. Research has demonstrated that IPL can help minimise clinical errors in patient care, leading to a higher level of satisfaction among both patients and health workers (Filies & Frantz 2021; Khanbodaghi et al. 2019; Sulaiman et al. 2021; Sunguya et al. 2014). One benefit of IPL is that it can break down the barriers between various professions and prompt shifts in attitudes, thereby reducing stereotypes among medical professionals (Kara et al. 2022). For students, IPL has been regarded as a favourable approach for developing an interest in patient care, transforming their outlook and improving their clinical and medical knowledge (Shakhman et al. 2020). Ensuring that patients receive optimal care and well-being requires coordinating treatments across professions from different viewpoints (Shakhman et al. 2020; Sunguya et al. 2014). One of the reasons for non-implementation may be how a clinician’s identity affects interprofessional collaborative practice. Nonetheless, several studies have shown that students in the health discipline have a positive attitude towards IPL (Alruwaili et al. 2020; Jimenez et al. 2018). According to Klein and Beeson (2022), opportunities for clinical mental health counsellors will likely increase as the larger healthcare system evolves towards more interprofessional healthcare settings. Although the counselling profession’s codes of ethics embed certain aspects of interprofessionalism, discussions on identity have primarily focused on interprofessional identity. To examine both intra- and interprofessional identity, a study was conducted with clinical mental health counsellors using the Professional Identity Scale in Counselling – Short Form and the University of West of England Interprofessional Questionnaire (Klein & Beeson 2022). The results revealed that clinical mental health counsellors assign importance to both types of identity, but they have more trust in their interprofessional identity (Klein & Beeson 2022). Graduate attribute studies in South Africa have found that teamwork among various disciplines is an essential competency for higher education institutions (Filies & Frantz 2021). Interprofessional learning instructors act as mentors, teachers and coaches for students from different professions, but they often feel underqualified because of their lack of necessary skills in dealing with diverse student populations with varying skill levels, responsibilities and social backgrounds. Researchers have started examining the culture, power dynamics, conflicts and systems present in practical contexts, which are believed to be responsible for IPL failures. These issues exacerbate the problem, as not only must IPL instructors teach interprofessional cooperation techniques in these environments, but they also must prepare students with tools to overcome obstacles that hinder their collaboration (Cimino et al. 2022; Wyatt, Kleinheksel & Tews 2021).

The efficacy of IPL is recognised by the WHO and included in the guidelines for transformative medical education (Aldriwesh, Alyousif & Alharbi 2022; Lee et al. 2019). To drive desired outcomes, countries are urged to promote IPL and incorporate it into their present curriculum. Namibia embarked on a curriculum transformation since 2021 in order to improve the graduate attributes such as ethical and moral leadership, global citizenry, problem-solving and critical thinking skills, adaptability and flexibility, effective communication skills, life-long learning and teamwork in all its undergraduate and postgraduate programmes. These attributes are a set of transferable characteristics designed to prepare them for the challenges of the 21st century workplace and society. Therefore, there is a need to investigate the readiness of students on IPL. This study assessed the readiness for IPL among undergraduate health discipline students at the University of Namibia.

## Theoretical framework

**FIGURE 1:**
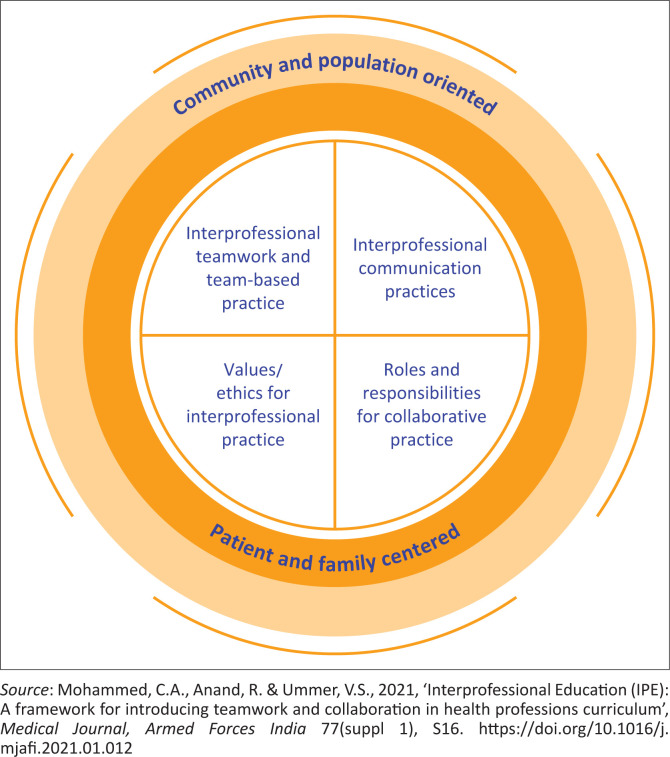
Framework for interprofessional education.

Interprofessional education promotes collaboration among students, which in turn improves the complex health needs of the population. In this study, four key competencies derived from the framework for interprofessional education were assessed (Mohammed, Anand & Ummer 2021). These competencies are values and ethics for interprofessional practice, roles and responsibilities for collaboration practice, interprofessional communication and teamwork (Figure 1). All of these competencies required collaboration. The values and ethics for interprofessional practice were assessed to determine how well individual students worked with others from different professions while maintaining a climate of mutual respect and shared values. The roles and responsibilities for collaboration practice were used to assess and address the healthcare needs of patients and promote the health of the population by using knowledge of one’s own role and responsibilities, as well as those of other professions. Interprofessional communication was assessed to determine how well individuals communicated with patients, families, communities and other healthcare professionals in a manner that supported a team approach to the promotion and maintenance of health and the prevention and treatment of diseases. The teamwork competency was to evaluate communication and teamwork skills as a team and also involved individual teamwork observation and feedback. These competencies provided a framework for both education and practice in this study. Nash (2023) claims that students’ roles and responsibilities would improve by giving opportunities to the healthcare team and learners to be involved in the shared clinical decision-making. Quality of care and patient outcomes would improve significantly if the healthcare workforce were trained based on the core competencies of interprofessional practice; hence, they are evaluated or assessed to build confidence when communicating and working together as a team (Mattheus & Roy 2022). Moreover, in this study, all four competencies were evaluated and assessed to attain the readiness for interprofessional learning among undergraduate healthcare discipline students.

## Research methods and design

### Design and setting

A quantitative descriptive research design was employed to evaluate the readiness for IPL among undergraduate health discipline students at a leading Namibian university. The cross-sectional descriptive design was chosen for its proven ability to accurately assess IPL readiness, aligning with the methodology utilised in comparable studies (Alruwaili et al. 2020; An et al. 2024; Kara et al. 2022).

The setting for this study encompassed three of the five health campuses at the University of Namibia, offering qualifications in health-related courses in Namibia. The selected campuses – Rundu, Hage Geingob and the Main Campus – serve an estimated population of 2000 undergraduate students across the School of Medicine, School of Dentistry, School of Pharmacy and School of Nursing and Public Health. These campuses are located in populated towns in Namibia, where two intermediate hospitals and one national referral hospital offer valuable opportunities for students in health disciplines to participate in practical attachments. A foundational premise of contemporary health education is that by the final year, health students must possess the capacity to comprehend, collaborate with and learn from peers and other members of the healthcare team to optimise patient outcomes.

### Population and sampling

The total population was 520 final-year undergraduate students enrolled in courses such as medicine, dentistry, pharmacy and nursing and public health at a leading university in Namibia. A total of 236 participants were recruited using purposive and random sampling. Sampling was conducted in two stages, beginning with the purposeful selection of sites, followed by random sampling to recruit the actual participants. The sample was calculated using Solvins’ formula (Equation 1):

$$n=\frac{N}{1+N*\alpha^{2}}$$ [Eqn 1]

where *n* is the sample size, *N* is the total population and α is the sampling error at 5% (Tejada & Punzalan 2012). To be eligible, participants were required to be final-year undergraduate students with access to the Internet and a prior collaborative experience, either through academic projects or during clinical placements. The study excluded participants who were sick, those with no Internet connectivity during the data collection period and those who were unwilling to participate.

### Measure

The study employed a validated online questionnaire tool, the readiness for interprofessional learning scale (RIPLS), developed from literature (Parsell & Bligh 1999; Roopnarine & Boeren 2020). The tool validated and consisted of 19 items in its three domains, namely teamwork and collaboration, professional identity and roles and responsibilities (McFadyen et al. 2005). This RIPLS tool embedded a domain, which collected demographic data, such as age, gender, religion, marital status and health discipline course. The instrument demonstrated strong internal reliability, achieving a Cronbach’s alpha (α) of 0.78, which surpasses the α > 0.70 standard (Taber 2018). Concurrently, the tool’s content validity was confirmed through review and approval by a panel of three specialist nurse educators.

*Teamwork and collaboration* is composed of nine items assessed through a 5-point Likert scale (1 = strongly disagree to 5 = strongly agree) (e.g. ‘Learning with other healthcare students will help me become a more effective member of a healthcare team’ and ‘Patients would ultimately benefit if healthcare students worked together to solve patient problems’).

*Professional identity was measured* with seven yes or no questions (e.g. ‘I don’t want to waste my time learning with other healthcare students’ and ‘It is not necessary for undergraduate healthcare students to learn together’).

*Roles and responsibilities* were assessed with three yes or no questions (e.g. ‘The function of nurses and therapists is mainly to provide support for doctors’ and ‘I have to acquire much more knowledge and skill than other healthcare students’). Importantly, negative statements (e.g. items 10, 11, 12 13 and 18) were scored in reverse such that a higher overall score indicates a higher readiness for IPL (McFadyen et al. 2005).

The total average score for this scale exhibits a range from a minimum of 19 to a maximum of 65. For the construct of readiness for teamwork and collaboration, a mean score within the range of 4.0 to 5.0 signifies a high degree of readiness. Conversely, a mean score of less than 4.0 indicates a low level of readiness. In contrast, the assessment of readiness towards professional identity and roles and responsibilities is evaluated using a different threshold. A mean score of ≥ 1.5 is indicative of greater readiness, while a mean scores of ≤ 1.4 suggest low readiness within this specific dimension.

### Data collection procedures

Data were collected between May and August 2023 to allow enough time to collect data from various campuses. The researcher’s involvement in the data collection process was limited to the dissemination of the questionnaire via participants’ academic WhatsApp groups. Participants completed the questionnaire on a self-administered basis. Two follow-up reminders were issued to encourage participation. During this phase, no clarifications, explanations or direct interactions occurred between the researcher and participants, ensuring that responses were not influenced. All questionnaires were completed anonymously. Only participants meeting the predefined inclusion criteria received the questionnaire. Electronic data collected were securely stored on the researcher’s password-protected personal computer.

### Data analysis

After downloading the responses on an Excel sheet from online Google Forms, the data were cleaned and coded before checking for completeness in order to exclude incorrect or missing data. Data were then analysed using SPSS version 28. To ensure consistent scoring, negative statements were reverse-scored prior to computing the mean value for the variable. Descriptive analysis was used by computing the mean scores and standard deviation of the constructs. A Spearman correlation test was used to explore the correlation between two ranked variables, given the abnormal data distribution, while the mean differences between health disciplines groups were assessed using a paired *t*-test. The regression model was used to predict factors for readiness at a 5% significance level.

### Ethical considerations

This study was granted ethical approval by the Ethics Committee of the School of Nursing and Public Health at the University of Namibia, under reference number SoN 43/2023. Prior to their involvement, all participants were provided with comprehensive information regarding the study’s aims, procedures, potential risks and benefits. Each participant then provided their written informed consent, demonstrating their voluntary agreement to take part in the research. Participants were explicitly informed of their right to withdraw from the study at any point, without consequence or loss of any benefits to which they were otherwise entitled. The research adhered strictly to the ethical principles delineated in the most recent Declaration of Helsinki, an internationally recognised standard for medical research involving human subjects. This compliance ensured the prevention of participant harm and was maintained by researchers possessing the requisite ethical and scientific expertise.

## Results

The survey was completed by a total of 236 participants, with a response rate of 100%. The majority of participants, 72% (*n* = 170), were from Nursing and Public Health, 8.9% (*n* = 21) from Dentistry, 12.3% (*n* = 29) from Medicine and 6.8% (*n* = 16) from Pharmacy. Most of the respondents were female, 59.3% (*n* = 140), followed by male, 40.7% (*n* = 96). A considerable portion of the respondents falls within the age range of 21 to 22 (48.7%; *n* = 115), followed by 23 to 27 year category (39%; *n* = 92), suggesting a relatively young population. Furthermore, the survey indicates that the majority of participants were single (95.8%; *n* = 226), while 4.2% (*n* = 10) were married (Table 1).

**TABLE 1:** Demographic characteristics.

Variable	Sub-variable	*n*	%
Age (years)	18–22	132	55.93
	23–27	92	39.00
	≥ 28	12	5.08
Gender	Female	140	59.30
	Male	96	40.70
Course of study	Dentistry	21	8.90
	Nursing and Public Health	170	72.00
	Medicine	29	12.30
	Pharmacy	16	6.80
Marital status	Single	226	95.80
	Married	10	4.20

### Mean readiness for interprofessional learning

Table 2 shows the results of each IPL subscale. The overall mean score readiness for the domain teamwork and collaboration skills was high at 4.22 ± 1.02. The area with the highest readiness were students’ readiness to work together to solve patient problems (4.50 ± 0.94), ready for shared learning with other healthcare students to understand clinical problems (4.37 ± 1.00), and learning with other healthcare students to become a more effective member of a healthcare team (4.35 ± 1.11). These findings highlight the importance of teamwork and collaboration in healthcare education and suggest that fostering these skills can lead to improved teamwork and patient outcomes.

**TABLE 2:** Interprofessional learning mean scores.

Item	Mean	s.d.
**Domain 1: Teamwork and collaboration**		
Learning with other healthcare students will help me become a more effective member of a healthcare team.	4.35	1.11
Patients would ultimately benefit if healthcare students worked together to solve patient problems.	4.50	0.94
Shared learning with other healthcare students will increase my ability to understand clinical problems.	4.37	1.00
Communication skills should be learned with other healthcare students	4.08	1.06
Teamworking skills are essential for all healthcare students to learn.	4.30	1.03
Shared learning will help me to understand my own limitations.	3.92	1.02
Learning with healthcare students before qualification would improve relationships after qualification.	4.04	1.02
Shared learning will help me to think positively about other professions.	4.12	1.01
For small group learning to work, students need to trust and respect each other.	4.33	0.98
Overall mean	4.22	1.02
**Domain 2: Professional identity satisfaction**		
I don’t want to waste my time learning with other healthcare students.	1.93	0.25
It is not necessary for undergraduate healthcare students to learn together.	1.83	0.37
Clinical problem-solving skills can only be learned with students from my own department.	1.83	0.38
Shared learning will help communicate better with patients and other professionals.	1.08	0.26
I would welcome the opportunity to work on small group projects with other healthcare students.	1.06	0.23
Shared learning will help to clarify the nature of patient problems.	1.04	0.20
Shared learning before qualification will help me become a better team worker.	1.06	0.23
Overall mean	1.40	0.27
**Domain 3: Roles and responsibilities**		
The roles of nurses and therapists are mainly to provide support to doctors.	1.40	0.49
I am not sure what my professional role will be.	1.83	0.37
I have to acquire much more knowledge and skills than other healthcare students.	1.41	0.49
Overall mean	1.54	0.45

s.d., standard deviation.

The results for the roles and responsibilities domain indicated high IPL readiness (1.54 ± 0.45), driven by a particularly high score for readiness in the professional role (1.83 ± 0.37). However, the overall professional identity readiness was low (1.40 ± 0.27), suggesting participants may not be fully prepared to articulate and execute their various responsibilities within a team setting.

### Correlation

The study aimed at assessing the relationship between domains of IPL and demographic characteristics. To achieve this, a Spearman’s *rho* correlation analysis was employed (Table 3). The study found a high positive correlation between professional identity and course of study (*rho* = 0.722; *p* = 0.010), followed by teamwork and collaboration and gender (*rho* = 0.650; *p* = 0.027) and teamwork and collaboration and marital status (*rho* = 0.641; *p* = 0.015). A moderate positive correlation was observed between teamwork and collaboration and health-related courses (*rho* = 0.537; *p* = 0.005) and professional identity and age (*rho* = 0.523; *p* = 0.002). No significant correlation was observed between teamwork and collaboration and age, as well as between course of study and roles and responsibilities (*p* > 0.05).

**TABLE 3:** Correlations between professional health disciplines (*p*-value).

Scale	Teamwork and collaboration	Professional identity	Roles and responsibilities
**Teamwork and collaboration skills**	1.000	-	-
**Professional identity satisfaction**	-	1.000	
*Rho*	0.278	-	-
*p*-value	0.048	-	-
**Roles and responsibilities**	-	-	1.000
*Rho*	0.391	0.234	-
*p*-value	0.051	0.059	-
**Age**	-	-	-
*Rho*	0.345	0.523	0.341
*p*-value	0.070	0.002	0.068
**Gender**	-	-	-
*Rho*	0.650	0.399	0.191
*p*-value	0.027	0.041	0.086
**Course of study**	-	-	-
*Rho*	0.537	0.722	0.303
*p*-value	0.005	0.010	0.097

### Comparison of readiness across health course disciplines

Table 4 presents a comparison of the readiness for IPL across various health disciplines. The domain with the highest average score was teamwork and collaboration across all health-related disciplines. The paired *t*-test results showed a highly statistically significant difference between teamwork and collaboration vs professional identity (*t* = 41.67) and teamwork and collaboration vs roles and responsibility (*t* = 39.42), for the Nursing and Public Health group, indicating the greatest magnitude of difference between these subscales in that group. No statistically significant mean difference (*p* = 0.110) was observed between professional identity and roles and responsibilities within the dentistry discipline.

**TABLE 4:** Comparison of Readiness for IPL Across Health Disciplines.

Discipline	Subscale comparison	Mean score	Standard deviation	*t*-value	*p*-value
Dentistry	Teamwork versus professional identity	4.33	1.10	15.12	< 0.001
	Teamwork versus roles and responsibility	4.33	1.57	13.09	< 0.001
	Professional identity versus roles and responsibility	1.10	1.57	1.67	0.110
Medicine	Professional identity versus professional identity	4.52	1.03	19.28	< 0.001
	Teamwork and collaboration versus roles and responsibility	4.52	1.59	21.60	< 0.001
	Professional identity versus roles and responsibility	1.03	1.59	6.22	< 0.001
Nursing and public health	Teamwork and collaboration versus professional identity	4.30	1.06	41.67	< 0.001
	Teamwork and collaboration versus roles and responsibility	4.30	1.61	39.42	< 0.001
	Professional identity versus roles and responsibility	1.06	1.61	5.19	< 0.001
Pharmacy	Teamwork and collaboration versus professional identity	4.56	1.19	25.98	< 0.001
	Teamwork and collaboration versus roles and responsibility	4.56	1.63	21.80	< 0.001
	Professional identity versus roles and responsibility	1.19	1.63	4.24	< 0.001

### Predictors of readiness for interprofessional learning

Table 5 delineates the predictive capacity of identified factors on readiness for IPL among health disciplines. In Model 1, work and collaboration emerged as the most robust predictor of readiness for IPL, accounting for a substantial 83.8% of the variance (*R* = 0.915; *R*^*2*^ = 0.838; *F* Change = 1208.751, *p* < 0.001). The 95% confidence interval, 95% CI (4.088–4.352), indicates a strong and statistically significant relationship, suggesting that increased engagement in work and collaboration is associated with a higher readiness for IPL.

**TABLE 5:** Predictors of interprofessional learning among health disciplines.

Predictors	Linear regression				Change statistics					95% CI	
	*R*	*R* ^2^	Adjusted *R*^2^	s.e. of the estimate	*R*^2^ change	*F* Change	*df* ^1^	*df* ^2^	Sig. *F* Change	Upper	Lower
Model 1*	0.915	0.838	0.837	0.117	0.838	1208.751	1	234	0.000	4.088	4.353
Model 2**	0.112	0.013	0.008	0.289	0.013	2.974	1	234	0.086	1.369	1.437
Model 3*	0.457	0.208	0.205	0.259	0.208	61.605	1	234	0.000	1.490	1.603

* , *p* < 0.01;

** , *p* < 0.10.

s.e., standard error; *df*, degrees of freedom; Sig., significant; CI, confidence interval.

Note: Dependent variable: readiness for interprofessional learning. Model 1: teamwork and collaboration; Model 2: professional identity; Model 3: roles and responsibilities.

Subsequently, Model 3 revealed that roles and responsibilities also served as a statistically significant predictor, explaining an additional 20.8% of the variance in readiness for IPL (*R* = 0.457; *R*^*2*^ = 0.208; *F* change = 61.605, *p* < 0.001). This indicates that clarity and understanding of roles and responsibilities significantly contribute to students’ readiness for IPL.

Conversely, Model 2 shows that professional identity did not demonstrate a statistically significant unique contribution to the variance in readiness for IPL (*R*^*2*^ = 0.013, *p* = 0.086), suggesting its individual predictive power was negligible within this analytical framework.

## Discussion

This study assessed and described the undergraduate health discipline students’ readiness for interprofessional learning using the framework for interprofessional education. The high mean scores observed in the current study for both teamwork and collaboration, and roles and responsibilities are consistent with a recent literature review by Patel et al. (2025). Literature suggests that introducing IPL during undergraduate training is a critical initial step towards alleviating the burden on healthcare systems (Carlisle & Taing 2021). However, when compared to the specific findings of previous individual studies (AlAhmari 2019; Kara et al. 2022; Vafadar, Vanaki & Ebadi 2015; Yasin et al. 2023), the present readiness levels are lower. The differences in results could be explained by students’ reluctance to collaborate with others, as well as by differences in knowledge on the benefits of collaboration with students from other health disciplines. In support of the differences, AlAhmari (2019) states that barriers that can hinder IPL readiness among healthcare professionals may be limited access to other healthcare streams, lack of adequate clinical training sites and scarce flexibility regarding curriculum requirements. These results underscore the importance of enhancing students’ preparedness for IPL and call for the identification and implementation of tailored strategies that align with the distinct academic disciplines represented by the students.

Despite the argument that diverse occupations can co-exist to enhance team success (Ahuja 2023), the current study found a low mean readiness for interprofessional identity. This may result in future professionals withholding information from their colleagues, thereby generating conflicts that hinder collaboration and the exchange of information. This finding aligns with Reinders and Krijnen (2023), who postulate that a low professional identity implies reduced interaction between different health disciplines. On the contrary, the recent study findings contradict An et al. (2024) study, which found a high mean score for the professional identity subscale. This difference in results could be attributed to the varying characteristics of the student groups surveyed.

The correlation analysis found a strong significant positive correlation between gender and teamwork and collaboration. This result indicates that as gender shifts towards female, the level of teamwork and collaboration tends to increase. Contrary to this result, Yasin et al. (2023) found no significant relationship between gender and teamwork (*p* = 0.297). Therefore, further studies need to investigate the role of gender in teamwork and collaboration.

Furthermore, a positive correlation was also observed between teamwork and collaboration and participants’ course of studies. It can be deduced that students were able to collaborate and team up with other healthcare students regardless of differences in their course of study to work effectively. Moreover, collaboration among other healthcare teams can provide an opportunity for one to become knowledgeable as they are able to explore different skills from others. Thus, these skills learned can be implemented and used to improve the care of patients. This is consistent with a prior study that reports a positive correlation (*r* = 0.508; *p* = 0.000) between health-related programmes and IPL (Stadick 2020). Atwa et al. (2023) study also found no significant differences between groups (*p* = 0.077).

There was a consistent, statistically significant difference when comparing teamwork and collaboration across dentistry, medicine, nursing and public health and pharmacy students. Two major predictors of IPL were identified. The first factor was teamwork, and collaboration was identified as the main predictor for IPL. Given a highly statistically significant mean score for teamwork, health discipline students are ready to collaborate and work with each other in fostering the care of patients’ needs in order to render quality care and improve patients’ health outcomes. These results are consistent with some previous studies (Amouzeshi, Daryazadeh & Keramatinejad 2023; Atwa et al. 2023; Jha et al. 2022), which reported a mean readiness score of 42.17 ± 2.83 for teamwork and collaboration. In line with the literature, the results of the current study prove that with effective collaboration, healthcare professionals can enhance their learning from each other, leading to improved collaboration in the workplace and higher quality measures for more efficient patient care services (AlAhmari 2019). Evidence indicates that the knowledge needed to work with other team members in a collaborative manner would be enriched and broadened by learning about the roles played by students in different professions, which has been associated with successful interprofessional practice (Soubra et al. 2018). Samarasekera et al. (2022) contend that the integration of teamwork and collaboration in health professional education can facilitate the synthesis of Western biomedicine and traditional Indigenous healthcare knowledge. This proposed integration is posited to lead to improved patient outcomes by fostering a more holistic and culturally sensitive approach to healthcare delivery. Moreover, teamwork and collaboration among healthcare workers have the potential to reduce errors, provide optimal treatment, utilise resources effectively and minimise costs (Atwa et al. 2023; Yasin et al. 2023). Given the increasing complexity of healthcare and the rising number of patients with intricate illnesses, it is crucial to foster a collaborative culture among healthcare workers to address complex patient issues. This culture can be successfully instilled during their time as students. A comparison of IPL readiness shows that nursing students had the highest mean readiness for teamwork and collaboration. This result is consistent with Mahboube et al.’s (2019) study, which claims that the attainment of favourable medical outcomes, as the utmost objective of the healthcare system, hinges primarily on the establishment of a productive rapport and harmonious cooperation among physicians and other team members, notably nurses. While doctors have a crucial role in the provision and coordination of care, nurses remain the backbone of the health system and the leading patient advocates (Shaban et al. 2024; Zhou, Li & Li 2021). These results demonstrate that nursing students have the awareness of their need to be instrumental in facilitating team efforts to improve patient care, as they are uniquely suited to serve as a clinical leader who will ensure that the team as a whole functions effectively and facilitates patient-centred decision-making.

Roles and responsibilities were identified as the second significant predictor of IPL readiness. This finding suggests that a student’s preparedness for IPL is directly influenced by their understanding and acceptance of their professional roles. Consistent with the findings of Nash (2023), it is implied that student readiness is likely strengthened through active participation in shared clinical decision-making. A clear understanding of one’s own professional role, as well as the roles of other interprofessional team members, is essential for effective collaboration. Success in IPL hinges on individuals first comprehending their own identity and the identities of others on the team (Wang et al. 2020). Research suggests that engaging in professional role exchange is a key mechanism for developing one’s identity. Such exchanges allow students to better understand others’ roles while gaining the critical ability to assess their own identity from an external viewpoint (Nyoni, Grobler & Botma 2021; Wang et al. 2020).

Interestingly, no statistically significant mean difference was observed between the professional identity and roles and responsibilities subscales for the dentistry student cohort. This suggests a lack of relationship between dentistry students’ attitudes towards their self-efficacy and mutual respect for tasks. This particular finding is consistent with results reported elsewhere in the literature. For instance, Ogbaghebriel and AlZeer (2021) documented insignificant findings for both professional identity (*p* = 0.441) and roles and responsibilities (*p* = 0.098), while Wong et al. (2023) specifically corroborated the non-significant mean difference (*p* = 0.349) concerning professional identity among dentistry students. Future research should be directed towards developing specific interventions designed to enhance domain-specific self-efficacy, role modelling, mentoring and professional socialisation among dentistry students in Namibia.

Clearly, the significant difference between teamwork and collaboration among students enrolled in various courses of study is suggestive that students can engage deeply with the material while also working alongside their peers. However, it is concerning that students may lack a clear sense of their professional role within a collaborative healthcare context. Future studies should prioritise interprofessional education initiatives to promote a robust interprofessional identity.

### Limitations

Despite yielding valuable data for the planning, development and review of health professional discipline curricula, this study is subject to several limitations. A primary limitation is the reliance on self-reported data, which introduces the potential for social desirability bias. Participants may have presented themselves in a more favourable light, aligning with perceived social norms and seeking to avoid negative evaluations regarding their readiness for IPL. To mitigate this bias, participants were explicitly informed about the importance of providing honest responses. Furthermore, the survey design allowed participants to complete the questionnaire in the comfort of their own homes, aiming to reduce external pressures and promote more candid disclosures.

For future research, a larger longitudinal study employing observational methods of IPL among health discipline students would provide more insightful data. Such an approach could offer a more robust basis for generalisation to similar educational settings and deliver a more comprehensive understanding of IPL readiness and development.

### Study contributions

The study’s findings directly contribute to practice, education and research. Given the significantly higher mean score for teamwork and collaboration, clinical settings are justified in immediately integrating newly graduated students into collaborative teams. For educators, the low professional identity readiness indicates a need to design specific, reflective interprofessional learning activities aimed at bolstering this core skill. Finally, research efforts must now be directed towards developing and evaluating targeted educational interventions to address the professional identity’s role in readiness.

## Conclusion

Despite a comparatively low mean readiness observed for professional identity, this study revealed a favourable inclination towards teamwork and collaboration and roles and responsibilities among health discipline students in Namibia. Consequently, teamwork and collaboration and roles and responsibilities emerged as the primary predictors of IPL within this cohort. This study significantly contributes to the existing framework for interprofessional education by identifying key findings pertinent to the assessment of IPL among health discipline students in Namibia. Specifically, it provides valuable insights into measurable aspects of IPL within this unique educational context. It is recommended that subsequent research focus on the strategic development of targeted interventions to enhance domain-specific self-efficacy among Namibian dentistry students.
